# A Treatise on the Cardinal Points of Dental Science and Practice

**Published:** 1847-10

**Authors:** 


### Bibliographical Notices.

*A Treatise on the Cardinal Points of Dental Science and Practice.* By S. M. Shepherd, Dental Surgeon, 24mo, pp. 20, Petersburg, Va. 1847.

The above little pamphlet is written in a plain and familiar style, and contains much valuable information. It is closely printed with small type, and is divided into eight chapters. The first treats upon the claims of dental science upon public attention, the attention the mouth requires during infancy for the preservation of the temporary teeth. The second is devoted to the management of first dentition, the importance of the deciduous teeth, the deleterious effects resulting from their premature loss, the means necessary for their preservation; the first four permanent molares. The third treats of the means necessary for the prevention of dental caries, and the causes of this disease. In the fourth, the curative indications of dental caries are pointed out, the causes of the prejudice entertained by the public against dental operations, and shows that the failures of dental operations to fulfil the end for which they were performed, are not attributable to any inefficiency of the resources of the art.

The fifth chapter treats of the agency of caries in the destruction of the teeth, effects of mercury and salivary calculi upon these organs, the use of the file upon them, and the prejudices that exist against the use of this instrument upon the teeth. Chapter six treats of the importance of filling teeth, the materials most proper to be employed for this operation, the impropriety of performing it after the nerve has become exposed, tooth-ache, and extraction as constituting the only sure remedy for the disease. The seventh chapter treats of the extirpation of the nerves of teeth, the morbid effects of diseased teeth, and the importance of preserving the health of the mouth. Chapter eight is devoted to artificial teeth, bad effects resulting from them when improperly applied, and their utility when properly constructed and inserted.—*Balt. Ed.*

